# Highly active N, S Co-Doped Ultramicroporous Carbon for High-Performance Supercapacitor Electrodes

**DOI:** 10.3390/mi13060905

**Published:** 2022-06-07

**Authors:** Wenjing Lu, Lina Hao, Yawei Wang

**Affiliations:** 1 College of Pharmacy, Suzhou Vocational Health College, Suzhou 215009, China; lnhao@szhct.edu.cn; 2 School of Chemistry and Chemical Engineering, Jiangxi Province Engineering Research Center of Ecological Chemical Industry, Jiujiang University, Jiujiang 332005, China

**Keywords:** carbon materials, energy storage and conversion, N, S co-doped, ultramicroporous, supercapacitors

## Abstract

N, S-doped ultramicroporous carbons (NSUC-x) with a high nitrogen/sulfur content and a narrow pore-size distribution of around 0.55 nm were firstly prepared using L-cysteine as a nitrogen and sulfur source. The phase, graphitization degree, morphology, specific surface area, pore structure and surface condition of NSUC-x are investigated to analyze the key role in electrochemical performance. Such an ultramicroporous structure and N, S doping not merely provide a high-specific surface area and a suitable pore size, but also induce a good wettability for the fast transport and adsorption of electrolyte ions. Due to the above strategies, the typical NSUC-0.4 exhibits a high gravimetric capacitance of 339 F g^−1^ at 0.5 A g^−1^ as well as a capacity retention of 91.6% after 10,000 cycles in a three-electrode system using a 6 M KOH electrolyte. More attractively, a NSUC-0.4-assembled symmetrical supercapacitor delivers an energy output of 7.4 Wh kg^−1^ at 100 W kg^−1^ in 6 M KOH as well as a capacity retention of 92.4% after 10,000 cycles, indicating its practical application prospect. Our findings open up new prospects for the design and electrochemical application of N, S-doped ultramicroporous carbons.

## 1. Introduction

With the fast economic development and the excessive consumption of fossil fuels, the energy dilemma and environmental pollution have made it essential to develop renewable and clean energy devices for a sustainable society [1,2,3]. As promising energy storage devices, supercapacitors have drawn increasing attention due to their merits of being low cost and having long-cycle stability, fast charge–discharge rates, superior power density to batteries and higher energy density than traditional capacitors [4,5,6]. Based on the energy storage mechanism, supercapacitors are commonly classified into two types: (1) electric double-layer capacitors (EDLCs) store energy by the electrostatic accumulation of charges at the electrode–electrolyte interfaces and (2) pseudo-capacitors store energy by fast Faradaic reactions between the electrode and electrolyte [7,8,9]. 

Carbon materials, including activated carbon, carbon nanotubes and carbon aerogels, are mainly applied as EDLC electrodes due to their high specific surface area and designable pore structure [10,11,12,13]. For microporous carbons, the <1 nm pores have a relationship with an anomalous increase in electrochemical performance [14,15]. Moreover, owing to the small hydrated radius of K^+^ (3.3 Å), Na^+^ (3.6 Å), OH^−^ (3.0 Å) and SO_4_^2^^−^ (3.8 Å), the >0.5 nm micropores are usually considered to be electrochemically available in aqueous electrolytes [16,17,18]. Bandosz et al. used palm date pits as precursors to prepare ultramicroporous carbon with a regular pore size of around 0.55 nm and a capacitance of 114 F g^−1^ at 1 mV s^−1^ in 6 M KOH [19]. They proved that not only <2 nm pores govern the electrochemical performance in aqueous electrolytes, but also that the differences in the distribution of the micropores are crucial. Apart from the surface area and pore structure, the surface condition of carbons also plays a determinant role in the electrochemical performance. Heteroatom (N, S, B, P, etc.) doping could modify the surface wettability and optimize the accessible surface of carbons [20,21,22]. In addition, the functional atoms on the surface could generate an additional pseudocapacitance due to fast and reversible Faradic reactions. Heteroatoms-doped carbons are usually prepared by the direct carbonization of precursors with a high content of heteroatoms or the high-temperature treatment of carbon in certain chemical environments (NH_3_, H_2_S, melamine, etc.) [23,24,25]. Unfortunately, the application of post-treatment is limited by the demerits of low heteroatom content, expensive cost of post-treatment reagents and pore blockage/collapse [26]. For example, Zhou et al. prepared N, O dual-doped carbons using diamino-anthraquinone as an N source, which revealed a high capacitance of 317 F g^−1^ at 0.5 A g^−1^ [27]. Via the self-doped approach, Ni et al. designed carbon foams with a 5.34 wt.% of N content for the supercapacitor electrodes, which exhibited a high capacitance of 273 F g^−1^ at 0.5 A g^−1^ in a 6 M KOH aqueous electrolyte [28]. 

In this paper, an N, S co-doped ultramicroporous carbon is synthesized by using L-cysteine as a nitrogen and sulfur source and exhibits a high specific capacitance of 339 F g^−1^ at a current density of 0.5 A g^−1^ (three-electrode system), as well as 7.4 Wh kg^−1^ at 100 W kg^−1^ (two-electrode system) in 6 M KOH with excellent cycling stability. These findings highlight that carbon materials with ultramicroporous structure and heteroatoms doping can be widely applied as high-performance supercapacitor electrodes.

## 2. Experimental Procedure

### 2.1. Synthesis of NSUC-x

All chemicals were used as received without any purification. In a typical synthesis, 0.126 g of phloroglucinol, 0.1 g of terephthalaldehyde, 0.2 mL ammonia solution and appropriate L-cysteine (0.3–0.5 g) were added to a 56 mL ethanol solution (28.6 vol.%) with stirring at 70 °C for 24 h. Subsequently, the mixed solution was transferred into a Teflon-lined autoclave and placed in an oven at 100 °C for 24 h to obtain the polymerized precursor. After washing in distilled water and alcohol, the polymer was dried in an oven at 60 °C for 48 h and carbonized at 600 °C for 4 h under pure N_2_ with a heating rate of 1 °C min^−1^. The resultant carbons were named nitrogen and sulfur co-doped ultramicroporous carbons (NSUC-x), in which x represents the mass of L-cysteine (g).

### 2.2. Characterization of NSUC-x

X-ray diffraction (XRD) patterns for characterizing the phase constitutions were tested on Bruker D8 Advance diffractometer (Germany) with the 2θ range of 10–80°. Raman spectra were recorded on a Renishaw Invia-spectrometer (UK) using a laser beam of 532.3 nm as the excitation source. After degassing at 200 °C for 2 h, the nitrogen sorption of all samples was measured at −196 °C by Micromeritics ASAP 2460 (Norcross, GA, USA). The Brunauer–Emmett–Teller (BET) model was applied to calculate the specific surface area, and the Density Functional Theory (DFT) model was used to estimate the pore parameters [29]. Surface conditions were investigated by an X-ray photoelectron spectrometer (XPS, AXIS Ultra DLD, Warwick, UK) equipped with an Al Kα radiation. Microstructures were observed by an S-4800 scanning electron microscope (SEM, Hitachi, Japan) and JEM-2100 transmission electron microscope (TEM, JEOL, Tokyo, Japan). The contact angle of water droplet was conducted using a Dataphysics DCAT21 instrument (Filderstadt, Germany). 

### 2.3. Electrochemical Characterization

A CHI660D electrochemical workstation (Shanghai Chenhua, China) was used to test all the electrochemical measurements. In a typical three-electrode system conducted in 6 M KOH electrolyte, Hg/HgO electrode and a Pt foil electrode were used as reference and counter electrodes, respectively. The working electrodes were fabricated by mixing 80 wt.% carbon materials, 10 wt.% polytetrafluoroethylene (PTFE) and 10 wt.% graphite, pressing on nickel foam and dried at 60 °C for 24 h. A voltage range from −1 to 0 V was applied in cyclic voltammetry (CV) and galvanostatic charge–discharge (GCD) tests, whose specific capacitance (*C*, F g^−1^) were calculated according to the Formulas (1) and (2), respectively:

(1) $$C=\frac{\int IdV}{2\Delta Vmr}$$

(2) $$C=\frac{I\Delta t}{\Delta Vm}$$

Moreover, a coin cell (NEWARE CR2032) was assembled with two identical NSUC-0.4 electrodes, 6 M KOH electrolyte, a piece of filter paper separator to analyze the two-electrode symmetric system. The capacitance of a single electrode (*C_two_*, F g^−1^), the energy densities (*E*, Wh kg^−1^) and power densities (*P*, W kg^−1^) of the device were obtained according to Formulas (3)–(5):

(3) $$C_{two}=4C_{device}=\frac{4I\Delta t}{\Delta Vm}$$

(4) $$E=\frac{C_{device}\Delta V^{2}}{7.2}$$

(5) $$P=\frac{3600E}{\Delta t}$$

In Formulas (1)–(5), *m* (g) is the mass of all active materials, Δ*V* (V) is the operation potential window, *Δt* (s) is the discharging current time, *r* (V s^−1^) is the scan rate and *I* (A) is the response current (Formula (1)) and the discharging current (Formulas (2) and (3)).

## 3. Results and Discussion

**Figure 1:**
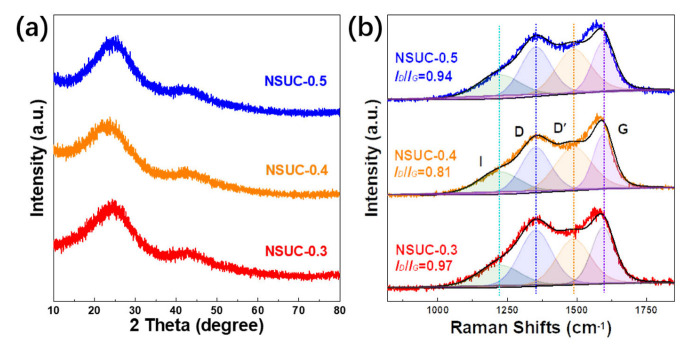
(**a**) XRD patterns and (**b**) Raman spectra of NSUC-x.

In Figure 1a, the XRD patterns of NSUC-*x* in the range from 10 to 80° exhibit two broad diffraction peaks around 23 and 44°, which are ascribed to the (002) and (001) lattice facets of amorphous carbon [30]. The graphitization degrees of all samples were further detected by Raman analysis. As depicted in Figure 1b, all spectra are deconvoluted into four Gaussian–Lorentzian peaks according to the literature [31,32]. Such four peaks around 1222, 1353, 1489 and 1595 cm^−1^ correspond to I band (caused by the impurities near carbon atoms), D band (derived from the defects/disorders in sp3 structure), D′ band (ascribed to the defects from stacked graphene layers) and G band (attributed to the vibration of graphitic sp^2^-type carbon), respectively [33,34]. The intensity ratio of the D band to G band *(I_D_/I_G_*) reflects the indicator of concrete graphitization degree. Apparently, the relative *I_D_/I_G_* in NSUC-0.4 is the lowest value (0.81) among all samples and implies the highest graphitization degree, which has a positive feedback on electronic conductivity. 

**Figure 2:**
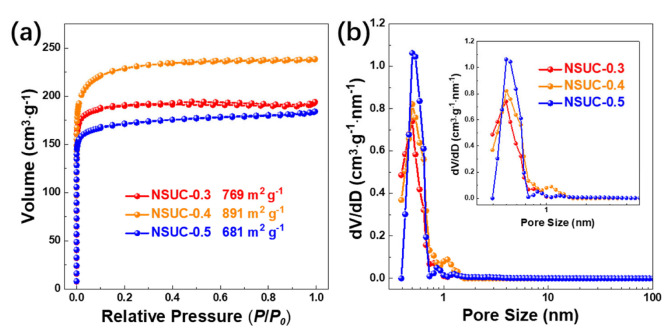
(**a**) N_2_ physisorption isotherms and (**b**) pore size distributions of NSUC-x.

The N_2_ physisorption isotherms of NSUC-x presented in Figure 2a belong to the type I curve according to the classification of IUPAC. As noted in all isotherms, the steep increase at low relative pressure (*P*/*P_0_* < 0.1) and the flat plateau (0.1 ≤ *P*/*P_0_* ≤ 1.0) are ascribed to abundant micropores. Moreover, the pore size distribution curves of NSUC-x depicted in Figure 2b exhibit unimodal and sharp peaks around 0.55 nm, indicating the presence of a highly ultramicroporous structure. As listed in Table 1 to compare the porous structure parameters, the largest surface area (891 m^2^ g^−1^) is achieved by NSUC-0.4, which also has the largest microporous specific surface area and total pore volume. The addition of L-cysteine has an important influence on the specific surface area of NSUC-x. When L-cysteine is not sufficient in the reaction, phloroglucinol cannot react with L-cysteine thoroughly, resulting in a decrement in the crosslinking density between phloroglucinol and L-cysteine. The region with a low crosslinking density pyrolyzes into gaseous products and leads to the collapse of the carbon matrix, resulting in the lower specific surface area in NSUC-0.3. On the otherwise, increasing the mass of *L*-cysteine beyond the optimum value increases the contents of N and S in the carbon samples. Due to the lower covalent band energy of C–N compared with C–C, the release of abundant nitrogen and sulfur atoms during the carbonization results in the partial collapse of the carbon framework, thus leading to the decrement in the specific surface area in NSUC-0.5. 

**Figure 3:**
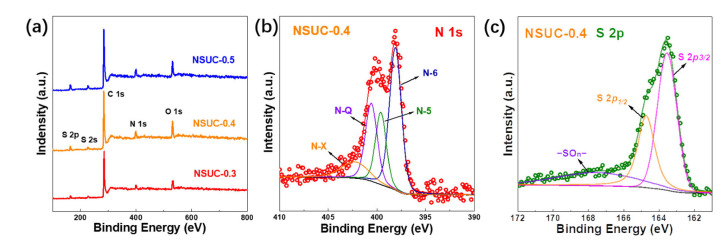
(**a**) XPS spectra of NSUC-x. Fitted high-solution XPS spectra of (**b**) N 1s and (**c**) S 2p for NSUC-0.4.

The surface chemical conditions of NSUC-x were investigated by XPS techniques. All spectra presented in Figure 3a exhibit signals for the binding energies of C 1*s*, O 1*s*, N 1*s* and S 2*p*, indicating the presence of four elements: C, N, S and O [35]. As tabulated in Table 1, the N and S content increased from 4.91 and 2.14 at.% to 8.11 and 3.01 at.% as the addition of L-cysteine increased. N 1*s* state involves four overlapping peaks of pyridine nitrogen oxide (N-X), graphitic nitrogen (N-Q), pyrrolic N (N-5) and pyridinic nitrogen (N-6). Usually, the N-5 with good electro–donor characteristics and high charge mobility is considered to enhance carbon catalytic activity in electron-transfer reactions and thus effectively improves electrochemical performance [36]. Meanwhile, the N−6 provides a pair of electrons for conjugation with the π-conjugated rings and thus can bring in electron–donor properties to carbon materials and efficiently enhance capacitances [37]. The N−Q can significantly boost the electron transfer and improve the conductivity of nitrogen-doping carbon materials [38]. The signals for S 2*p* state can be identified as three resolved peaks at 163.5 eV, 164.7 eV and 167.4 eV, being ascribed to S 2p*_3/2_*, S 2p*_1/2_* and oxidized sulfur moiety (−SO_n_−). The two prominent peaks correspond to the S 2p*_3/2_* and S 2p*_1/2_* of the C–S–C covalent bond of the thiophene-S caused by spin-orbit coupling [39]. N, S co-doping can comprehensively improve the electrochemical performance of carbon electrodes by optimizing the surface condition, electronic conductivity and Faradaic reactivity.

**Figure 4:**
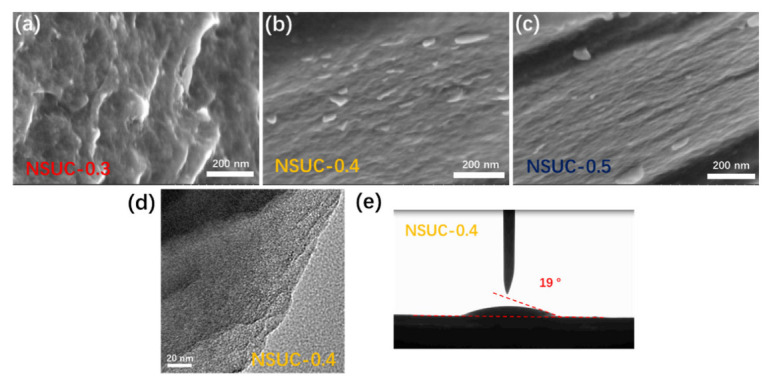
(**a**–**c**) SEM images of NSUC-x, (**d**) TEM images of NSUC-0.4, and (**e**) the contact angle of water on the surface of NSUC-0.4.

All morphologies of NSUC-x observed in SEM images (Figure 4a–c) show a large bulk structure with a coarse surface, which becomes smooth with the increasing addition of L-cysteine. The morphology and porous structure of NSUC-0.4 were further investigated by the TEM method (Figure 4d). It can be noted that NSUC-0.4 shows a disordered ultramicroporous structure, which is consistent with the above pore size distribution. As shown in Figure 4e, NSUC-0.4 shows a small water contact angle of 19°, implying that heteroatom doping can ameliorate the surface hydrophilicity of the carbons [40]. In this regard, the aqueous electrolyte ions have a good pore accessibility in the NSUC-0.4 electrode and can readily saturate into the inner surface, facilitating the electrochemical capacitance performance. 

**Figure 5:**
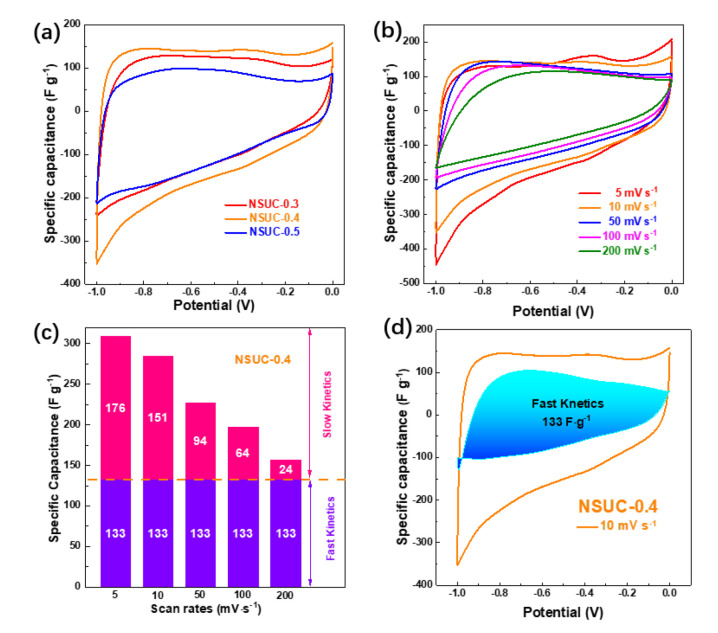
(**a**) CV curves at 10 mV s^−1^, (**b**) CV curves of NSUC-0.4 at different scan rates, (**c**) histogram of capacitance contribution and (**d**) decoupling capacitance from slow-kinetic processes (blank) and fast-kinetic processes (blue region).

The CV curves of NSUC-x in Figure 5a exhibit a quasi-rectangular shape with a deformation, indicating the EDLC and pseudocapacitance energy storage. As displayed, the capacitance of NSUC-0.4 calculated from CV curves is much higher than those of the other two electrodes. For the NSUC-0.4 electrode in Figure 5b, an unobvious current hump occurs at −0.37 V as the scan rate decreases to 5 mV s^−1^, indicating the presence of pseudocapacitance induced by N, S co-doping. The capacitive contribution of NSUC-0.4 (Figure 5c,d) was decoupled as the equation of $i=k_{1}v+k_{2}v^{1/2}$, where *k_1_v* and *k_2_v^1/2^* equal to the current density associated with fast- and slow-kinetic process [41,42]. The fast-kinetic capacitance of NSUC-0.4 is 133 F g^−1^, which is mainly contributed to EDLC. 

**Figure 6:**
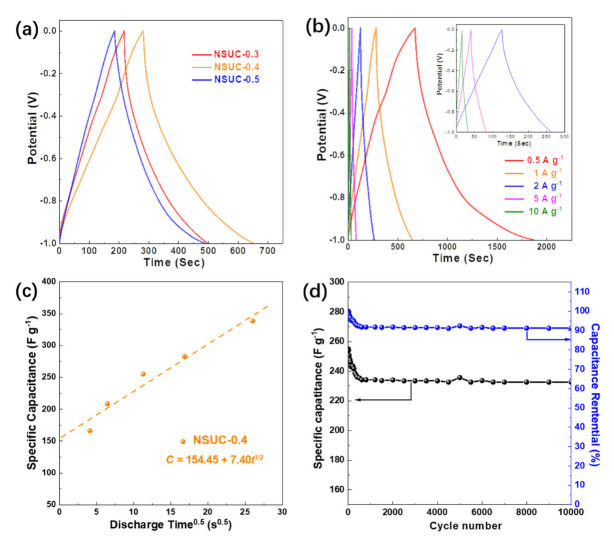
(**a**) GCD curves at 1 A g^−1^, (**b**) GCD curves of NSUC-0.4 at different current densities, (**c**) capacitance of NSUC-0.4 versus the square root of discharge time and (**d**) cyclic stability of NSUC-0.4 at 2 A g^−1^.

The GCD profiles of NSUC-x at 1.0 A g^−1^ are shown in Figure 6a. In the manner of CV curves, all GCD profiles exhibit a quasi-triangular distribution with a little distortion, implying satisfactory electrochemical capacitive properties with a pseudocapacitive procedure. Meanwhile, the specific capacitance (283 F g^−1^) of the NSUC-0.4 electrode integrated from GCD is dramatically higher than those of NSUC-0.3 (218 F g^−1^) and NSUC-0.5 (189 F g^−1^). In Figure 6b, NSUC-0.4 exhibits an ultrahigh capacitance of 339 F g^−1^ at 0.5 A g^−1^ as well as remains 189 F g^−1^ with a triangle shape up to 10 A g^−1^, signaling a high-rate charge–discharge capability. The charging–discharging kinetics of NSUC-0.4 were also discussed according to the formula $C_{T}=C_{E}+kt^{1/2}$, where *C_T_*, *C_E_* and *t* are the total capacitance, EDLC and discharging time, respectively [43,44]. As shown in Figure 6c, the *C_E_* of NSUC-0.4 is 155 F g^−1^ and close to the value (133 F g^−1^) fitted by CV curves. Thereby, the ultramicropores and N, S doping, which mainly contributed to the slow-kinetic capacitance, are crucial to the enhancement of the electrochemical energy storage. The 10,000 consecutive charge–discharge cycles in Figure 6d were tested on the NSUC-0.4 electrode with a capacity retention of 91.6% at 2 A g^−1^, indicating an excellent cycling stability. The electrochemical performance of NSUC-0.4 is compared with that of other carbon materials in Table 2.

**Figure 7:**
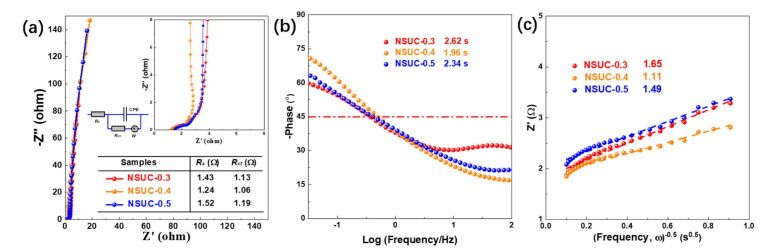
(**a**) Nyquist plots, (**b**) Bode plots and (**c**) Randles plots of NSUC-x.

Nyquist plots of NSUC-x electrodes measured in 6 M KOH aqueous solution in a frequency range of 0.01 to 10^5^ Hz are shown in Figure 7a. Obviously, NSUC-0.4 electrode not only exhibits the equivalent series resistance (R_s_, 1.24 Ω) and the charge-transfer resistance (R_ct_, 1.06 Ω) with the smallest quasi-semicircle in the high-frequency region. Abundant micropores, especially ultramicropores, can benefit the fast transportation and diffusion of electrolyte ions to enhance the electrochemical performance. At the same time, the existence of nitrogen and sulfur elements can improve the surface properties and electric conductivity. Meanwhile, the relaxation time (τ) and the diffusive resistance (σ) of NSUC-0.4 obtained from Figure 7b,c are 1.96 s and 1.11 Ω s^−1/2^, respectively, which were lower than those of NSUC-0.3 and NSUC-0.5 [54]. These ESR, τ and σ values imply the low resistance and the fast electrolyte/ion diffusion achieved by the NSUC-0.4 electrode.

**Figure 8:**
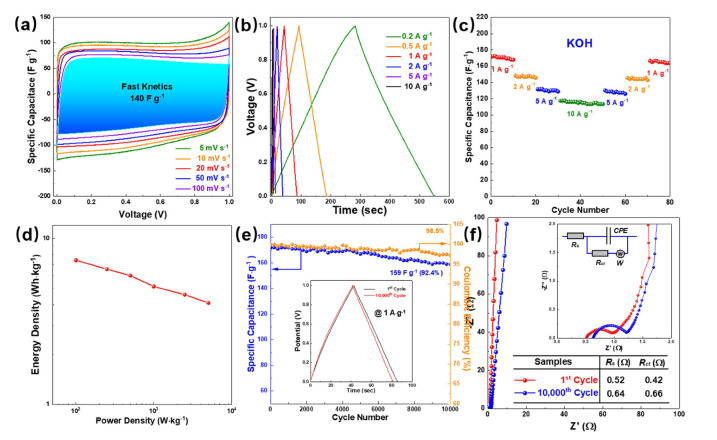
Electrochemical performance of NSUC-0.4 measured in a two-electrode system using a 6 M KOH as electrolyte: (**a**) CV curves at different scan rates, (**b**) GCD curves at different current densities, (**c**) rate performance, (**d**) Ragone plots, (**e**) cycling stability at 1 A g^−1^ (the inset shows the 1st cycle and 10,000th cycle GCD curves) and (**f**) Nyquist plots.

Figure 8 shows the electrochemical performance of NSUC-0.4 measured in two-electrode system using a 6 M KOH as electrolyte. In Figure 8a, all curves from 5 to 100 mV s^−1^ exhibit the rectangular shapes, implying the rapid and reversible charge–discharge procedure. Moreover, GCD curves detected with 0.2 to 10 A g^−1^ (Figure 8b) show symmetrical triangle shapes, further confirming the EDLC performance. The rate performance in Figure 8c demonstrates the high reversibility of the NSUC-0.4 electrode. As shown in the Ragone plots in Figure 8d, the KOH-loaded device displays a high energy density of 7.4 Wh kg^−1^ at 100 W kg^−1^ with excellent long cycle life (retention of 92.4%) and coulombic efficiency (98.5%) after 10,000 cycles (Figure 8e). Nyquist plots in Figure 8f further compared the resistance of the NSUC-0.4 electrode before and after 10,000 cycles. Both of the plots are nearly parallel to the imaginary axis in the low-frequency range, 45° diagonal lines in the intermediate frequency region and semicircles in the high-frequency area. The R_s_ (0.64 Ω) and R_ct_ (0.66 Ω) of the 10,000th cycle are slightly larger than those of the 1^st^ cycle (R_s_ = 0.52 Ω and R_ct_ = 0.42 Ω), meaning a low electrochemical resistance in the KOH-loaded device with a good stability.

## 4. Conclusions

N, S co-doped ultramicroporous carbons were fabricated by applying the L-cysteine as a nitrogen and sulfur source. The high nitrogen (7.35 at.%)/sulfur (2.37 at.%) content as well as the ultramicroporous structure enhance the electrochemical performance of NSUC-0.4 in the following aspects: (i) heteroatom doping provides additional pseudocapacitance and superior hydrophilic surface, and (ii) the superior hydrophilic surface and high graphitization degree make the high specific surface area (891 m^2^ g^−1^) to be effectively employed in electrochemical processes. Thereby, NSUC-0.4 exhibits an excellent specific capacitance of 339 F g^−1^ at 0.5 A g^−1^. The assembled symmetrical supercapacitor based on the NSUC-0.4 electrode delivers an energy output of 7.4 Wh kg^−1^ at 100 W kg^−1^ in 6 M KOH with a long cycle life. Our results firmly show a new avenue that the heteroatoms-doped ultramicroporous carbons provide for advanced energy storage devices with a high electrochemical performance. 

## Figures and Tables

**Table 1:** Pore structure parameters and chemical compositions of NSUC-*x*.

Samples	Pore Structure Parameters			Element Content (at.%)			
	*S_BET_*	*S_Micro_*	*V_tol_*	C	N	S	O
NSUC-0.3	769	701	0.30	85.85	4.91	2.14	7.11
NSUC-0.4	891	789	0.37	80.13	7.35	2.37	10.15
NSUC-0.5	681	598	0.28	80.43	8.11	3.01	8.44

*S_BET_*: specific surface area (m^2^ g^−1^); *S_Micro_*: micropore surface area (m^2^ g^−1^); *V_tol_*: total pore volume (cm^3^ g^−1^).

**Table 2:** Comparison of the electrochemical performances of the reported carbon-based electrodes in three-electrode system.

Sample	Heteroatoms	Electrolyte	Voltage (V)	Capacitance (F g^−1^)	Ref.
NSUC-0.4	N, S	6 M KOH	−1–0	339 (0.5 A g^−1^) 283 (1 A g^−1^)	This work
Hydrochar	N, S	6 M KOH	−1–0	227.3 (1 A g^−1^)	[45]
HBFC-1	N, O	2 M KOH	−1–0	194.5 (0.5 A g^−1^)	[46]
Thr-C_0.1_-B	B, N	6 M KOH	−1–0	242 (1 A g^−1^)	[35]
S@G5	S	6 M KOH	−1–0	257 (0.25 A g^−1^)	[47]
PPC900-N&P30	N, P	2 M KOH	−1–0	240 (0.5 A g^−1^)	[48]
NSC	N, S	6 M KOH	−1–0	288 (0.5 A g^−1^)	[49]
N_2_PC_5_	N, O	6 M KOH	−1–0	321.5 (0.5 A g^−1^)	[50]
NSPC-600	N, S	6 M KOH	−1–0	358 (0.5 A g^−1^)	[51]
NOC_700,1:1_	N, O	1 M H_2_SO_4_	−0.2–0.8	311 (1 A g^−1^)	[40]
PSC_800_	N, O	6 M KOH	−1–0	344 (1 A g^−1^)	[52]
NCM-700	N, O	6 M KOH	−1–0	228 (1 A g^−1^)	[53]

## Data Availability

Not applicable.

## References

[1] Miao L., Song Z., Zhu D., Li L., Gan L., Liu M. (2021). Ionic Liquids for Supercapacitive Energy Storage: A Mini-Review. Energy Fuel.

[2] Zhou X.L., Zhang H., Shao L.M., Lü F., He P.J. (2021). Preparation and Application of Hierarchical Porous Carbon Materials from Waste and Biomass: A Review. Waste Biomass Valoriz..

[3] Shao Y., El-Kady M.F., Sun J., Li Y., Zhang Q., Zhu M., Wang H., Dunn B., Kaner R.B. (2018). Design and Mechanisms of Asymmetric Supercapacitors. Chem. Rev..

[4] Simon P., Gogotsi Y. (2020). Perspectives for electrochemical capacitors and related devices. Nat. Mater..

[5] Raza W., Ali F.Z., Raza N., Luo Y.W., Kim K.H., Yang J.H., Kumar S., Mehmood A., Kwon E.E. (2018). Recent advancements in supercapacitor technology. Nano Energy.

[6] Noori A., El-Kady M.F., Rahmanifar M.S., Kaner R.B., Mousavi M.F. (2019). Towards establishing standard performance metrics for batteries, supercapacitors and beyond. Chem. Soc. Rev..

[7] Zhu D.Z., Wang Y.W., Lu W.J., Zhang H., Song Z.Y., Luo D., Gan L.H., Liu M.X., Sun D.M. (2017). A novel synthesis of hierarchical porous carbons from interpenetrating polymer networks for high performance supercapacitor electrodes. Carbon.

[8] Miao L., Song Z.Y., Zhu D.Z., Li L.C., Gan L.H., Liu M.X. (2020). Recent advances in carbon-based supercapacitors. Mater. Adv..

[9] Ray A., Roy A., Ghosh M., Ramos-Ramon J.A., Saha S., Pal U., Bhattacharya S.K., Das S. (2019). Study on charge storage mechanism in working electrodes fabricated by sol-gel derived spinel NiMn2O4 nanoparticles for supercapacitor application. Appl. Surf. Sci..

[10] Song Z., Miao L., Li L., Zhu D., Gan L., Liu M. (2021). A robust strategy of solvent choice to synthesize optimal nanostructured carbon for efficient energy storage. Carbon.

[11] Lei C.C., Ji C.C., Mi H.Y., Yang C.C., Zhang Q., He S.X., Bai Z.Y., Qiu J.S. (2020). Engineering kinetics-favorable carbon sheets with an intrinsic network for a superior supercapacitor containing a dual cross-linked hydrogel electrolyte. ACS Appl. Mater. Inter..

[12] Liang T.T., Hou R.L., Dou Q.Y., Zhang H.Z., Yan X.B. (2021). The applications of water-in-salt electrolytes in electrochemical energy storage devices. Adv. Funct. Mater..

[13] Wang Y., Hao L., Zeng Y., Cao X., Huang H., Liu J., Chen X., Wei S., Gan L., Yang P. (2021). Three-dimensional hierarchical porous carbon derived from resorcinol formaldehyde-zinc tatrate/poly(styrene-maleic anhydride) for high performance supercapacitor electrode. J. Alloys Compd..

[14] Gu Y.Y., Miao L., Yin Y., Liu M.X., Gan L.H., Li L.C. (2021). Highly N/O co-doped ultramicroporous carbons derived from nonporous metal-organic framework for high performance supercapacitors. Chin. Chem. Lett..

[15] Miao L., Duan H., Liu M.X., Lu W.J., Zhu D.Z., Chen T., Li L.C., Gan L.H. (2017). Poly(ionic liquid)-derived, N, S-codoped ultramicroporous carbon nanoparticles for supercapacitors. Chem. Eng. J..

[16] Nightingale Jr E. (1959). Phenomenological theory of ion solvation. Effective radii of hydrated ions. J. Phys. Chem..

[17] Guardia L., Suarez L., Querejeta N., Vretenar V., Kotrusz P., Skakalova V., Centeno T.A. (2019). Biomass waste-carbon/reduced graphene oxide composite electrodes for enhanced supercapacitors. Electrochim. Acta.

[18] Xu B., Hou S.S., Duan H., Cao G.P., Chu M., Yang Y.S. (2013). Ultramicroporous carbon as electrode material for supercapacitors. J. Power Sources.

[19] Barczak M., Elsayed Y., Jagiello J., Bandosz T.J. (2018). Exploring the effect of ultramicropore distribution on gravimetric capacitance of nanoporous carbons. Electrochim. Acta.

[20] Ping Y.J., Yang S.J., Han J.Z., Li X., Zhang H.L., Xiong B.Y., Fang P.F., He C.Q. (2021). N-self-doped graphitic carbon aerogels derived from metal-organic frameworks as supercapacitor electrode materials with high-performance. Electrochim. Acta.

[21] Miao L., Zhu D.Z., Liu M.X., Duan H., Wang Z.W., Lv Y.K., Xiong W., Zhu Q.J., Li L.C., Chai X.L. (2018). N, S Co-doped hierarchical porous carbon rods derived from protic salt: Facile synthesis for high energy density supercapacitors. Electrochim. Acta.

[22] Liu M.Q., Huo S.L., Xu M., Wu L.L., Liu M.J., Xue Y.F., Yan Y.M. (2018). Structural engineering of N/S co-doped carbon material as high-performance electrode for supercapacitors. Electrochim. Acta.

[23] Laheaar A., Delpeux-Ouldriane S., Lust E., Beguin F. (2014). Ammonia Treatment of Activated Carbon Powders for Supercapacitor Electrode Application. J. Electrochem. Soc..

[24] Feng S.S., Li W., Shi Q., Li Y.H., Chen J.C., Ling Y., Asiri A.M., Zhao D.Y. (2014). Synthesis of nitrogen-doped hollow carbon nanospheres for CO_2_ capture. Chem. Commun..

[25] Kim J.W., Kim B., Park S.W., Kim W., Shim J.H. (2014). Atomic layer deposition of ruthenium on plasma-treated vertically aligned carbon nanotubes for high-performance ultracapacitors. Nanotechnology.

[26] Biener J., Stadermann M., Suss M., Worsley M.A., Biener M.M., Rose K.A., Baumann T.F. (2011). Advanced carbon aerogels for energy applications. Energy Environ. Sci..

[27] Zhou M., Li X.Y., Zhao H., Wang J., Zhao Y.P., Ge F.Y., Cai Z.S. (2018). Combined effect of nitrogen and oxygen heteroatoms and micropores of porous carbon frameworks from Schiff-base networks on their high supercapacitance. J. Mater. Chem. A.

[28] Ni L., Wang R.W., Wang H.B., Sun C.Y., Sun B., Guo X., Jiang S., Shi Z.Q., Jing W.D., Zhu L.K. (2018). Designing nanographitic domains in N-doped porous carbon foam for high performance supercapacitors. Carbon.

[29] Thommes M., Kaneko K., Neimark A.V., Olivier J.P., Rodriguez-Reinoso F., Rouquerol J., Sing K.S.W. (2015). Physisorption of gases, with special reference to the evaluation of surface area and pore size distribution (IUPAC Technical Report). Pure Appl. Chem..

[30] Liu J., Qiao S.Z., Liu H., Chen J., Orpe A., Zhao D.Y., Lu G.Q. (2011). Extension of The Stober Method to the Preparation of Monodisperse Resorcinol-Formaldehyde Resin Polymer and Carbon Spheres. Angew. Chem. Int. Edit..

[31] Ferrari A.C., Robertson J. (2000). Interpretation of Raman spectra of disordered and amorphous carbon. Phys. Rev. B.

[32] Ferrari A.C., Basko D.M. (2013). Raman spectroscopy as a versatile tool for studying the properties of graphene. Nat. Nanotechnol..

[33] Wu X.K., Yu M., Liu J.H., Li S.M., Zhang X.L. (2020). sp^3^-Defect and pore engineered carbon framework for high energy density supercapacitors. J. Power Sources.

[34] Qian X.Y., Miao L., Jiang J.X., Ping G.C., Xiong W., Lv Y.K., Liu Y.F., Gan L.H., Zhua D.Z., Liu M.X. (2020). Hydrangea-like N/O codoped porous carbons for high-energy supercapacitors. Chem. Eng. J..

[35] Wang H., Zhou H., Wu S.M., Li Z.G., Fan B.B., Li Y.H., Zhou Y.M. (2021). Facile synthesis of N/B co-doped hierarchically porous carbon materials based on threonine protic ionic liquids for supercapacitor. Electrochim. Acta.

[36] Yang Y., Liu Y.X., Li Y., Deng B.W., Yin B., Yang M.B. (2020). Design of compressible and elastic N-doped porous carbon nanofiber aerogels as binder-free supercapacitor electrodes. J. Mater. Chem. A.

[37] Wickramaratne N.P., Xu J.T., Wang M., Zhu L., Dai L.M., Jaroniec M. (2014). Nitrogen Enriched Porous Carbon Spheres: Attractive Materials for Supercapacitor Electrodes and CO_2_ Adsorption. Chem. Mater..

[38] Lv H., Wang S.F., Xiao Z.Y., Qin C.R., Zhai S.R., Wang G.X., Zhao Z.Y., An Q.D. (2021). N-doped hollow carbon spheres with controllable shell numbers for high-performance electrical double-layer capacitors. J. Power Sources.

[39] Zhou J., Shen H.L., Li Z.H., Zhang S., Zhao Y.T., Bi X., Wang Y.S., Cui H.Y., Zhuo S.P. (2016). Porous carbon materials with dual N, S-doping and uniform ultra-microporosity for high performance supercapacitors. Electrochim. Acta.

[40] Zhou Z.Y., Miao L., Duan H., Wang Z.W., Lv Y.K., Xiong W., Zhu D.Z., Li L.C., Liu M.X., Gan L.H. (2020). Highly active N, O-doped hierarchical porous carbons for high-energy supercapacitors. Chin. Chem. Lett..

[41] Song Z.Y., Miao L., Ruhlmann L., Lv Y.K., Zhu D.Z., Li L.C., Gan L.H., Liu M.X. (2021). Self-assembled carbon superstructures achieving ultra-stable and fast proton-coupled charge storage kinetics. Adv. Mater..

[42] Long C., Miao L., Zhu D., Duan H., Lv Y., Li L., Liu M., Gan L. (2021). Adapting a kinetics-enhanced carbon nanostructure to Li/Na hybrid water-in-salt electrolyte for high-energy aqueous supercapacitors. ACS Appl. Energy Mater..

[43] Li Z.W., Chen D.H., An Y.F., Chen C.L., Wu L.Y., Chen Z.J., Sun Y., Zhang X.G. (2020). Flexible and anti-freezing quasi-solid-state zinc ion hybrid supercapacitors based on pencil shavings derived porous carbon. Energy Storage Mater..

[44] Wang H., Wang M., Tang Y.B. (2018). A novel zinc-ion hybrid supercapacitor for long-life and low-cost energy storage applications. Energy Storage Mater..

[45] Shan Y.Q., Xu Z.X., Duan P.G., Fan H.L., Hu X., Luque R. (2020). Nitrogen- and Sulfur-Doped Carbon Obtained from Direct Hydrothermal Carbonization of Cellulose and Ammonium Sulfate for Supercapacitor Applications. ACS Sustain. Chem. Eng..

[46] Hamouda H.A., Cui S.Z., Dai X.W., Xiao L.L., Xie X., Peng H., Ma G.F. (2021). Synthesis of porous carbon material based on biomass derived from hibiscus sabdariffa fruits as active electrodes for high-performance symmetric supercapacitors. RSC Adv..

[47] Kan Y.F., Ning G.Q., Ma X.L. (2017). Sulfur-decorated nanomesh graphene for high-performance supercapacitors. Chin. Chem. Lett..

[48] Wang Z., Tan Y.T., Yang Y.L., Zhao X.N., Liu Y., Niu L.Y., Tichnell B., Kong L.B., Kang L., Liu Z. (2018). Pomelo peels-derived porous activated carbon microsheets dual-doped with nitrogen and phosphorus for high performance electrochemical capacitors. J. Power Sources.

[49] Song P., He X.M., Shen X.P., Sun Y.M., Li Z.W., Yuan A.H., Zhai L.Z., Zhang D.Y. (2019). Dissolution-assistant all-in-one synthesis of N and S dual-doped porous carbon for high-performance supercapacitors. Adv. Powder Technol..

[50] Nie Z.G., Wang Y., Li X.Y., Wang R., Zhao Y., Song H., Wang H. (2021). Heteroatom-doped hierarchical porous carbon from corn straw for high-performance supercapacitor. J. Energy Storage.

[51] Liang X.D., Liu R.N., Wu X.L. (2021). Biomass waste derived functionalized hierarchical porous carbon with high gravimetric and volumetric capacitances for supercapacitors. Micropor. Mesopor. Mater..

[52] Xue D.F., Zhu D.Z., Xiong W., Cao T.C., Wang Z.W., Lv Y.K., Li L.C., Liu M.X., Gan L.H. (2019). Template-free, self-doped approach to porous carbon spheres with high N/O contents for high-performance supercapacitors. ACS Sustain. Chem. Eng..

[53] Zhu D.Z., Wang Y.W., Gan L.H., Liu M.X., Cheng K., Zhao Y.H., Deng X.X., Sun D.M. (2015). Nitrogen-containing carbon microspheres for supercapacitor electrodes. Electrochim. Acta.

[54] Song Z.Y., Miao L., Li L.C., Zhu D.Z., Lv Y.K., Xiong W., Duan H., Wang Z.W., Gan L.H., Liu M.X. (2020). A universal strategy to obtain highly redox-active porous carbons for efficient energy storage. J. Mater. Chem. A.

